# Targeted alpha therapy for chronic lymphocytic leukaemia and non-Hodgkin’s lymphoma with the anti-CD37 radioimmunoconjugate ^212^Pb-NNV003

**DOI:** 10.1371/journal.pone.0230526

**Published:** 2020-03-18

**Authors:** Astri Fjelde Maaland, Amal Saidi, Julien Torgue, Helen Heyerdahl, Tania A. Rozgaja Stallons, Arne Kolstad, Jostein Dahle

**Affiliations:** 1 Nordic Nanovector ASA, Oslo, Norway; 2 Institute of Clinical Medicine, University of Oslo, Oslo, Norway; 3 Orano Med SAS, Courbevoie, France; 4 Orano Med LLC, Plano, Texas, United States of America; 5 Department of Oncology, Oslo University Hospital, Radiumhospitalet, Oslo, Norway; 6 KG Jebsen Center for Cancer Immunotherapy, Institute of Clinical Medicine, University of Oslo, Oslo, Norway; University of Wisconsin, UNITED STATES

## Abstract

Relapse of chronic lymphocytic leukaemia and non-Hodgkin’s lymphoma after standard of care treatment is common and new therapies are needed. The targeted alpha therapy with ^212^Pb-NNV003 presented in this study combines cytotoxic α-particles from ^212^Pb, with the anti-CD37 antibody NNV003, targeting B-cell malignancies. The goal of this study was to explore ^212^Pb-NNV003 for treatment of CD37 positive chronic lymphocytic leukaemia and non-Hodgkin’s lymphoma in preclinical mouse models.An anti-proliferative effect of ^212^Pb-NNV003 was observed in both chronic lymphocytic leukaemia (MEC-2) and Burkitt’s lymphoma (Daudi) cells *in vitro*. In biodistribution experiments, accumulation of ^212^Pb-NNV003 was 23%ID/g and 16%ID/g in Daudi and MEC-2 tumours 24 h post injection. In two intravenous animal models 90% of the mice treated with a single injection of ^212^Pb-NNV003 were alive 28 weeks post cell injection. Median survival times of control groups were 5–9 weeks. There was no significant difference between different specific activities of ^212^Pb-NNV003 with regards to therapeutic effect or toxicity. For therapeutically effective activities, a transient haematological toxicity was observed. This study shows that ^212^Pb-NNV003 is effective and safe in preclinical models of CD37 positive chronic lymphocytic leukaemia and non-Hodgkin’s lymphoma, warranting future clinical testing.

## Introduction

In the USA, chronic lymphocytic leukaemia (CLL) and non-Hodgkin’s lymphoma (NHL) account for 1.2% and 4.3% of all new cancer incidence, with a combined estimated number of new cases of approximately 95,000 in 2019 [1, 2]. The standard of care for CLL is chemotherapy in combination with anti-CD20 antibodies. However, small molecular inhibitors are emerging as new therapies. While these regimens are initially effective in inducing responses, most patients eventually relapse and become refractory to further treatments [3–5]. NHL comprises a more heterogeneous group of diseases where treatment varies between subtypes. The backbone for most patients is chemotherapy combined with anti-CD20 antibodies. Indolent types of NHL are commonly diagnosed at advanced stage and thus incurable, but median survival is expected to be 15–20 years [6]. More aggressive subtypes of NHL are curable with intensive therapies, but patients who experience relapse often have a dismal outcome [7]. Both for CLL and NHL, new therapies with different mechanisms of actions and targets are needed.

In this study we explore a novel strategy, a targeted alpha therapy (TAT). We have conjugated the IgG1 chimeric antibody NNV003 with the chelator TCMC and labelled it with the alpha-particle generating radionuclide ^212^Pb (^212^Pb-NNV003). NNV003 binds with high affinity to CD37 and has been shown to internalise in some cell lines and induce antibody-dependent cellular phagocytosis and antibody-dependent cellular cytotoxicity [8]. CD37 is a glycosylated transmembrane protein, which has emerged as a therapeutic target in the recent years. It is highly and selectively expressed by B-lymphocytes and B-cell malignancies [9]. There are currently three CD37 targeting therapies in clinical development [10–13]. One of these compounds, ^177^Lu-lilotomab satetraxetan (Betalutin®), applies the β-emitter lutetium-177 as the cytotoxic payload, and is currently in clinical phase 2b for patients with relapsed follicular lymphoma (NCT01796171) [13]. Unlike NHL, where enlarged lymph nodes and tumours dominate the clinical picture, CLL more often presents as a disseminated leukemic disease. In theory, it would be more advantageous to use an α-emitter as the cytotoxic payload in CLL. Due to the α-particles’ short range of 50–100 μm, the radiation will be more localised to target cells than β-particles that have a range of 0.05–12 mm. The α-particles’ high LET of 100 keV/μm creates irreparable DNA double strand breaks. Consequently, only 2–3 α-particles are needed to kill a single cell, compared to 100–1000 low LET β-particles [14]. ^212^Pb has two alternative decay pathways through α-emitting daughters, ^212^Bi or ^212^Po, and can therefore be used as an *in vivo* generator of α-particles [15].

The anti-tumour efficacy of ^212^Pb has been demonstrated in preclinical studies; in several animal models of peritoneal cancer [16–22], prostate cancer, melanoma, pancreatic cancer and breast cancer [23–26]. It has also been applied in a pre-targeting setting [27, 28]. Recently, a phase 1 trial with ^212^Pb-TCMC-trastuzumab documented safety and feasibility in patients with human HER2 expressing malignancies [29]. Furthermore, a phase 1 study of ^212^Pb-DOTAMTATE for treatment of neuroendocrine tumours has been initiated (NCT03466216). In our study, we have investigated the *in vitro* cytotoxic effect of ^212^Pb-NNV003 in a CLL and a Burkitt’s lymphoma cell line. The *in vivo* tumour targeting of the TAT was studied in subcutaneous xenograft models. Two different disseminated models of CLL and NHL were used to evaluate the *in vivo* anti-tumour efficacy and toxicity of ^212^Pb-NNV003.

## Materials and methods

### Ethics statement–animal research

All studies were conducted under the approval of the institutional IACUC committee, Orano Med Institutional Animal Care and Use Committee, ethical approval number IAC-PR-006. Mice were kept under pathogen-free condition in a 12-hour light/dark cycle, with ad libitum access to food and water. Temperature, humidity and air-flow was continuously monitored. The cages contained enrichments and the bedding was changed once a week. Animal health was monitored by trained staff. The mice were euthanised by cervical dislocation when humane end point was reached. ARRIVE guidelines were followed (S2 File). See Supplementary S1 File and S1 Table for more information.

### Labelling antibodies with ^212^Pb

NNV003 and cetuximab (binding to EGFR, used as unspecific control, Merck KGaA, Germany) were conjugated with a customised bifunctional version of TCMC (1,4,7,10-Tetrakis(carbamoylmethyl)-1,4,7,10-tetraazacyclododecane, Macrocyclics, USA), using an enzymatic procedure based on a process described by Jeger [30] and Dennler [31] resulting in up to two TCMC molecules conjugated to a specific amino acid in the Fc part of the antibody. An over 99.9% radiochemically pure ^212^Pb was eluted with 0.4 M ammonium acetate from a ^224^Ra generator (Orano Med LLC, USA). TCMC-NNV003 and TCMC-cetuximab in 150 mM ammonium acetate were added to purified ^212^Pb at ratios of 3.7, 37 or 370 MBq/mg and incubated at 37°C for 10 min with shaking at 300 rpm. ITLC was used to confirm a chelation > 95%. Samples were diluted in 0.9% NaCl prior to injection. Specific activities (SA) used: 37 MBq/mg (biodistribution and acute toxicity studies), 370 MBq/mg (cytotoxicity assay) and 3.7–370 MBq/mg (therapy studies). The immunoreactivity (IRF) of ^212^Pb-NNV003 was measured as previously described [8].

### Cell lines

The human CLL cell line MEC-2 (Creative Bioarray, USA) and the Burkitt’s lymphoma cell line Daudi (ATCC, USA) were cultured in IMDM and RPMI medium. Media were supplemented with 10% heat inactivated fetal bovine serum and 1% Penicillin-Streptomycin (ATCC, USA).

### *In vitro* studies

MEC-2 and Daudi cells (1x10^6^) were fixed in 1% formaldehyde for 15 min at 4°C, stained with 5 μL Alexa Fluor® 647 mouse anti-human CD37 (Clone M-B371, BD Bioscience, USA) for 30 min on ice in the dark and analysed in a Guava easyCyte 8HT (Millipore, USA).

MEC-2 and Daudi cells were plated in 96 well-plates with 5000 cells/well. ^212^Pb-NNV003 or ^212^Pb-cetuximab was added to the cells at final concentrations of 57.8 Bq/ml to 14.8 kBq/ml (n = 8 wells per concentration). The cells were incubated for 5 h before washing. After resuspending in fresh medium, the cells were kept in culture for 6 more days. The CyQUANT™ NF Cell Proliferation Assay Kit (Thermo Fisher Scientific, USA) was used to measure cell proliferation.

### Biodistribution

10x10^6^ Daudi or 2.5x10^6^ MEC-2 cells were injected subcutaneously (s.c) in the flank of 15 female CB17 SCID mice (CB17/Icr-*Prkdc*^*scid*^/IcrIcoCrl, Charles River Laboratories, USA) or 24 female R2G2 mice (B6;129-*Rag2*^*tm1Fwa*^*II2rg*^*tm1Rsky*^/DwlHsd, Envigo, USA). When tumours reached a volume of 200–300 mm^3^, 200 μg murine IgG2a (M7769-5MG, Sigma Aldrich, USA) was injected intraperitoneally (i.p.). Next day, 370 kBq ^212^Pb-NNV003 was injected intravenously (i.v.). Mice were euthanised at predetermined time-points: 1 h (n = 5 CB17 SCID, n = 10 R2G2), 6 h (n = 5 CB17 SCID, n = 4 R2G2) and 24 h (n = 5 CB17 SCID, n = 10 R2G2). Organs and tumours were harvested, weighted and the activity was measured by a calibrated gamma counter (Wizard2, Perkin Elmer, USA). The background was subtracted from the measurements and values were decay corrected. Percent injected dose/g (%ID/g) was calculated for each tissue.

### Radiation dosimetry

The biodistribution data from the two s.c. models was used to calculate the absorbed radiation doses from ^212^Pb-NNV003, performed by Rapid (Maryland, USA). Time-integrated activity coefficients were obtained by the trapezoidal method as the data could not be exponentially fitted. Physical decay was used to extrapolate after the last time point.

### Toxicity studies of ^212^Pb-NNV003

Female CB17 SCID mice (30 total, n = 5 per group) were injected i.v. with ^212^Pb-NNV003 or PBS. Female R2G2 mice (29 total) were injected i.v. with ^212^Pb-NNV003 (n = 5 per group), 0.9% NaCl or ^212^Pb-cetuximab (n = 3 per group), to ensure similar tolerability of the two TATs. 200 μg murine IgG2a was injected i.p. one day before TAT injection. The mice were weighed three times a week and observed daily for clinical signs of radiotoxicity. Mice were euthanised when termination criteria were met (see Termination criteria section). Histopathological examinations were performed by Comparative Bioscience Inc (USA) on organs collected from R2G2 mice.

In both therapy models described below the concentration of platelets, red blood cells and white blood cells were monitored (see Haematological toxicity section).

### Therapy studies

To mimic disseminated CLL disease, 68 female R2G2 mice were i.v. injected with 2.5x10^6^ MEC-2 cells two days prior to treatment with ^212^Pb-NNV003 (370 MBq/mg), ^212^Pb-cetuximab (370 MBq/mg), NNV003-TCMC or 0.9% NaCl (n = 10). This model was also used to test different SAs of ^212^Pb-NNV003. 70 R2G2 mice were i.v. injected with MEC-2 cells, and received 370 kBq ^212^Pb-NNV003 (3.7, 37 or 370 MBq/mg), 370 kBq ^212^Pb-cetuximab (3.7 MBq/mg), NNV003-TCMC or 0.9% NaCl (n = 10 per group).

67 female CB17 SCID mice were i.v. injected with 10x10^6^ Daudi cells two days before treatment with ^212^Pb-NNV003 (370 MBq/mg), ^212^Pb-cetuximab, NNV003-TCMC or 0.9% NaCl (n = 12 for 280 kBq ^212^Pb-NNV003 and n = 11 for the other groups). In all studies, animals received 200 μg murine IgG2a i.p. the day before treatment. The mice were checked daily for clinical symptoms and body weights were monitored. They were euthanised when termination criteria were met (see Termination criteria section). Statistical analysis performed as described in Statistics section.

### Haematological toxicity

In both therapy models in the study, the concentration of platelets, red blood cells and white blood cells were monitored. 100 μL blood was collected prior to treatment and every two weeks thereafter from the retro-orbital sinus. The cell concentrations were determined using Vetscan HM5 hematology analyzer (Abaxis, USA). Animals received 300 μL 0.9% NaCl i.p. after blood collection. Statistical analysis was performed as described in the Statistics section.

### Termination criteria

Animals were euthanised by cervical dislocation when a combination of following humane end-points occurred: weight loss > 15% over two consecutive days, lack of grooming over 5 days, weakness over 3 days, reduced motility, paralysis, palpable abdominal tumour > 1000 mm^3^, hunched back, severe anaemia and diarrhoea.

### Statistics

All statistical analysis were done in GraphPad Prism 7.00 (GraphPad Software, USA). Log rank tests were performed for pairwise comparisons of treatment groups in the therapy studies. The Holm-Sidak method for multiple comparisons correction was used, with a significance level of α = 0.05. The platelet counts of the ^212^Pb-NNV003 treated mice were compared with the NaCl treated mice by one-way ANOVA followed by the Dunnett’s multiple comparison test with significance level of α = 0.05.

## Results

### Immunoreactivity of ^212^Pb-NNV003

The IRF of ^212^Pb-NNV003 was measured after initiation of the studies and was found to be around 57%. This suboptimal binding was due to radiation induced oxidation of the antibody after labelling and not the conjugation method. The addition of ascorbic acid during labelling restored the binding of ^212^Pb-NNV003 to the cells to approximately 80%, which is normally obtained with NNV003 labelled with lutetium-177 [8].

### CD37 expression and cytotoxicity of ^212^Pb-NNV003

**Fig 1:**
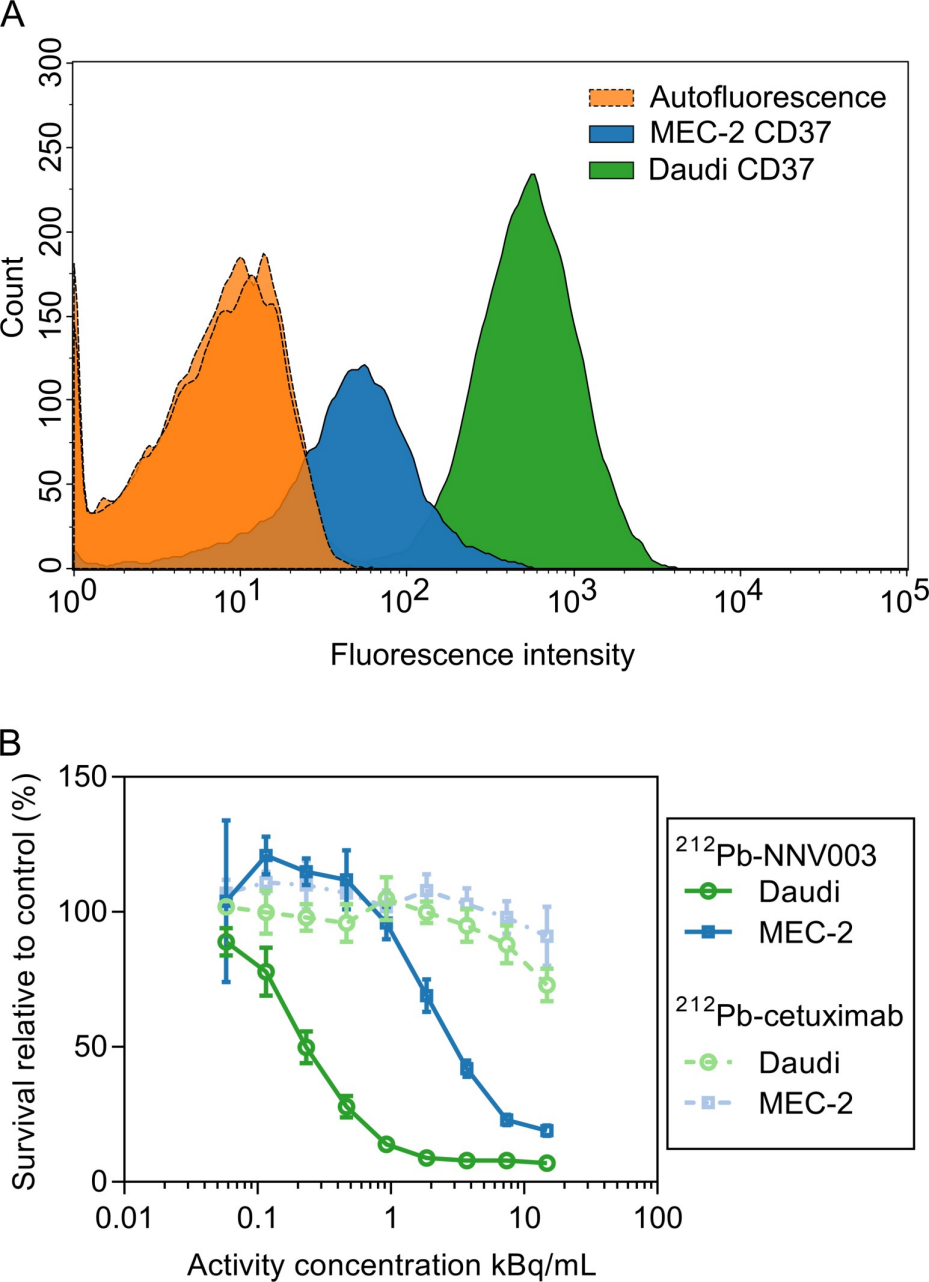
CD37 expression and cytotoxic effect of ^212^Pb-NNV003. (A) Flow cytometry histograms of cells only and cells incubated with 5 μl Alexa Fluor® 647 anti-human CD37. (B) Proliferation of Daudi and MEC-2 cells treated with ^212^Pb-NNV003 or ^212^Pb-cetuximab. Data represented as average of n = 8 replicates and error bars = SD.

CD37 expression was approximately 20 times higher in Daudi cells than MEC-2 cells (Fig 1A). ^212^Pb-NNV003 had a dose dependent anti-proliferative effect on both cell lines, while ^212^Pb-cetuximab only had modest effect at the highest concentrations (Fig 1B). Daudi cells appeared more sensitive than MEC-2 cells. The experiment was repeated, and the trend was confirmed (S2 Fig).

### Biodistribution and dosimetry of ^212^Pb-NNV003

In each animal study the mice were predosed with murine IgG2a before TAT injection to decrease the binding of ^212^Pb-NNV003 to murine Fc receptors and thus prevent clearance of antibody to spleen and liver in immune deficient mice with low amounts of endogenous antibodies [32]. Murine IgG2a binds with a similar affinity as human IgG1 to murine Fc receptors [33, 34]. A biodistribution performed in CB17 SCID mice revealed significant decrease in ^212^Pb-NNV003 uptake in spleen, kidneys and liver (S3A Fig). In immune competent Balb/c mice, however, the biodistribution was not altered by the predosing with IgG2a (S3B Fig).

**Fig 2:**
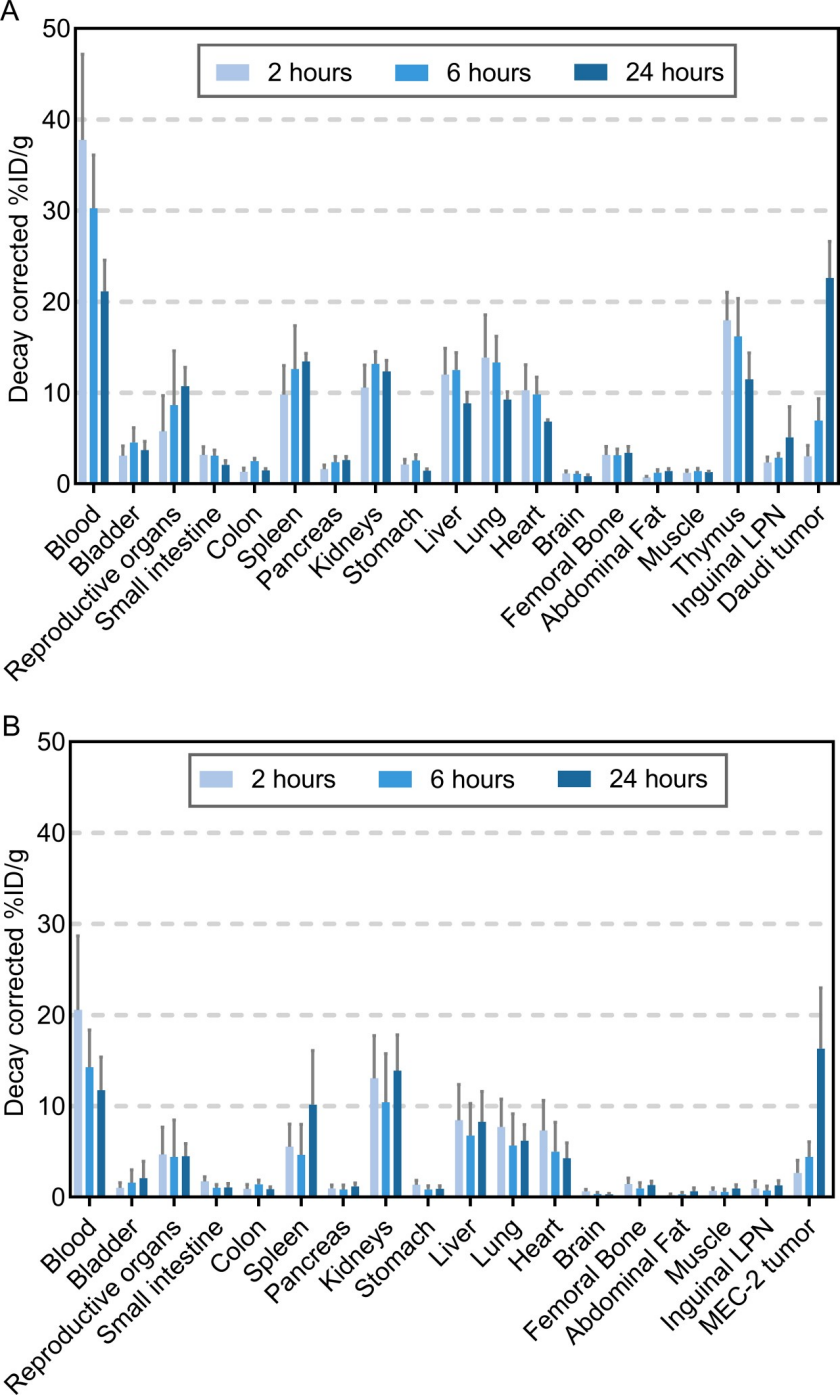
Biodistribution of ^212^Pb-NNV003. %ID/g of ^212^Pb-NNV003 in tissues of (A) CB17 SCID mice with Daudi s.c. xenografts (n = 5 per time point) and (B) R2G2 mice with MEC-2 s.c. xenografts (n = 4 at 6 h, n = 10 at 2 and 24 h). Data presented as averages with error bars = SD, LPN = Lymph Node.

^212^Pb-NNV003 was rapidly taken up in blood rich organs and thymus. Accumulation in tumour was slower but reached approximately 23%ID/g in Daudi tumours and 16%ID/g in MEC-2 tumours after 24 h (Fig 2A and 2B). The lack of redistribution of the radionuclide after initial uptake in organs indicates *in vivo* stability of ^212^Pb-NNV003.

**Fig 3:**
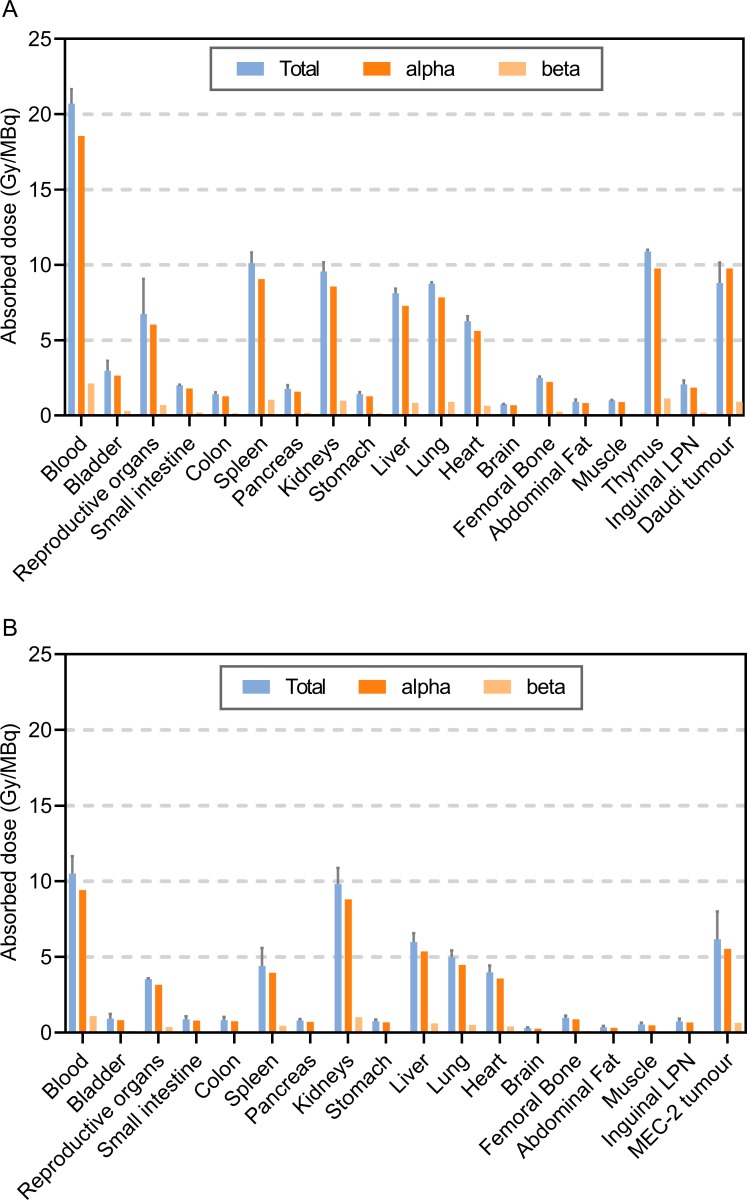
Dosimetry of ^212^Pb-NNV003. Absorbed radiation dose (Gy/MBq) to tissues of (A) CB17 SCID mice with Daudi s.c. xenografts (n = 5 per time point) and (B) R2G2 mice with MEC-2 s.c. xenografts (n = 4 at 6 h, n = 10 at 2 and 24 h). Error bars = SD of total absorbed radiation dose.

The tissue absorbed doses from the TAT is presented in Fig 3. Alpha radiation contributes most to the total absorbed dose, which was highest in blood rich organs and in the tumours.

### Acute toxicity of ^212^Pb-NNV003

The CB17 SCID mice that reached termination criteria were euthanised because of acute radiation toxicity. One mouse treated with 740 kBq was found dead in the cage, assumed dead of radiation toxicity. No R2G2 mice reached termination criteria. Remaining CB17 SCID and the R2G2 mice were euthanised at the end of the studies, 29 (CB17 SCID) or 33 (R2G2) days after TAT injection.

**Fig 4:**
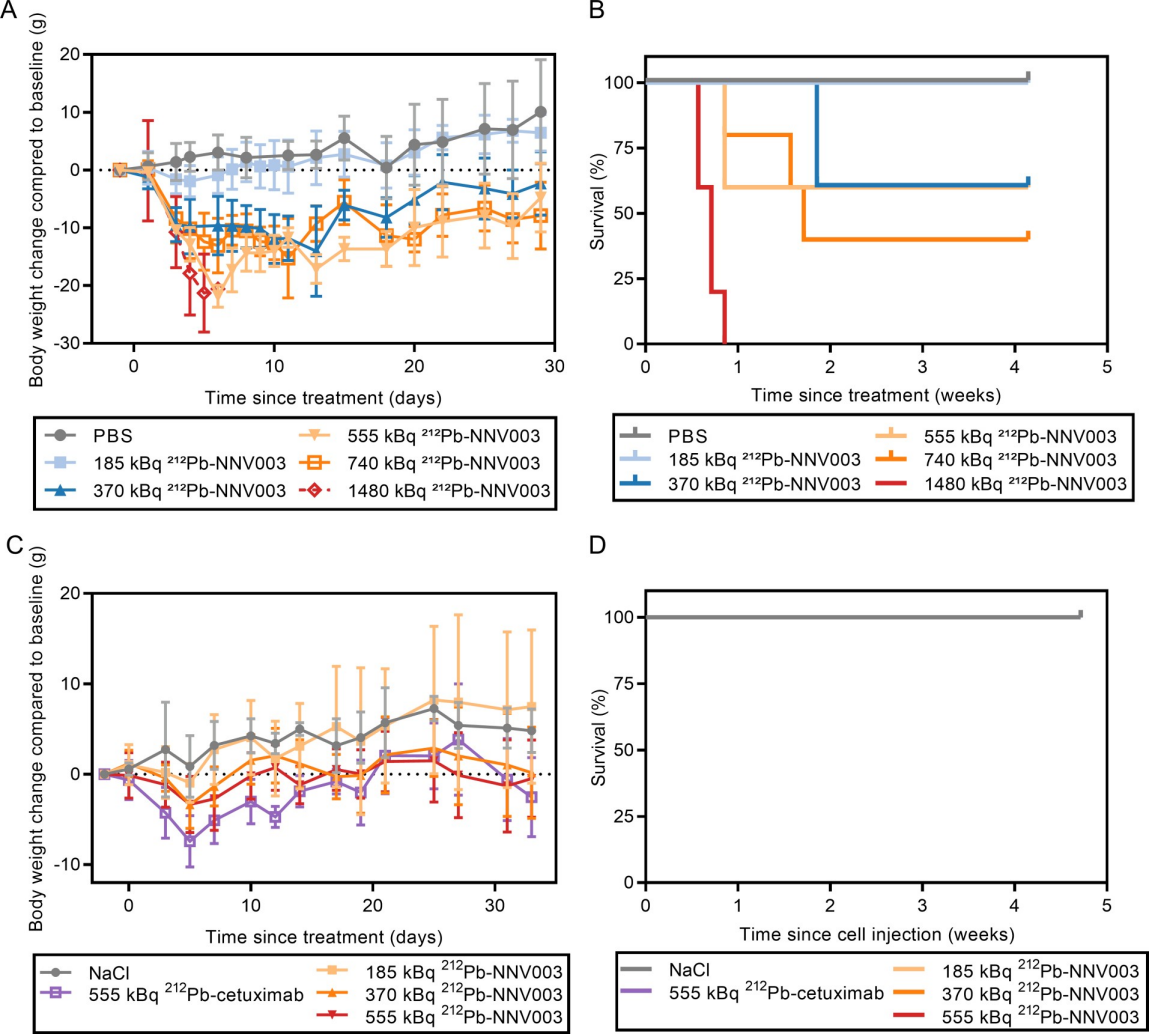
Acute toxicity of ^212^Pb-NNV003. CB17 SCIDs were injected i.v. with increasing dose of ^212^Pb-NNV003 or PBS. (A) Body weights (average of n = 5, error bars = SD) and (B) survival of the mice. R2G2 mice were injected with increasing dose of ^212^Pb-NNV003 (n = 5), ^212^Pb-cetuximab or NaCl (n = 3). (C) Body weight (average with error bars = SD) and (D) survival of the mice (overlapping curves).

1480 kBq ^212^Pb-NNV003 was too toxic for the CB17 SCID mice, and within a week after injection, all mice had been euthanised due to weight loss (Fig 4A and 4B). Doses of 370–740 kBq ^212^Pb-NNV003 also caused radiotoxicity with weight loss and 40–60% of the mice had to be euthanised within three weeks post injection. However, the lowest dose, 185 kBq, was well tolerated. CB17 SCID mice are known to have low tolerance to ionising radiation, due to a deficiency in the DNA double strand break repair mechanism [35]. Therefore, R2G2 mice were used for the MEC-2 model since they do not possess the SCID mutation and are therefore less sensitive to radiation. Indeed, doses of 185–555 kBq of ^212^Pb-NNV003 and 555 kBq of ^212^Pb-cetuximab could be administered in R2G2 mice without mortality, with only a mild and reversible initial weight loss (Fig 4C and 4D). Histopathological examination of the treated R2G2 mice showed no signs of radiation induced damage. From these results, the highest non-severely toxic doses (HNSTD) were established: 185 kBq in CB17 SCID mice and 555 kBq in R2G2 mice, and the following ^212^Pb-NNV003 doses were chosen for therapy studies: 90, 185 and 280 kBq (CB17 SCID) and 185, 370, 555 and 740 kBq (R2G2). The two additional doses of 280 kBq (CB17 SCID) and 740 kBq (R2G2) that were not tested in the acute toxicity studies were included to test the range of the therapeutic window.

### Anti-tumour effect of ^212^Pb-NNV003

**Fig 5:**
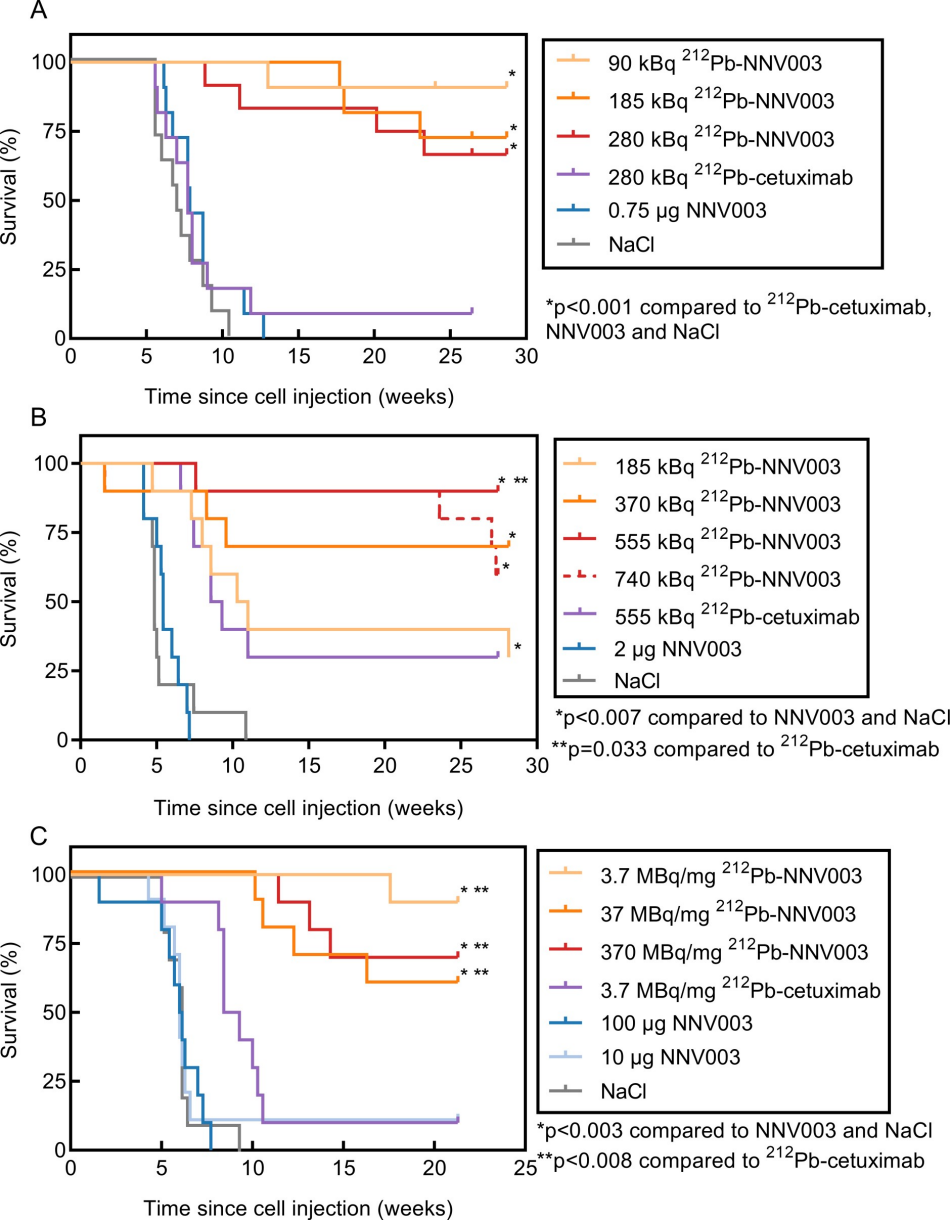
Anti-tumour effect of ^212^Pb-NNV003. Survival of (A) CB17 SCID mice (n = 11 or 12) i.v. injected with Daudi cells and of (B) R2G2 mice (n = 10) i.v. injected with MEC-2 cells two days prior to treatment with ^212^Pb-NNV003, ^212^Pb-cetuximab, NNV003 or NaCl. (C) Survival of R2G2 mice (n = 10) i.v. injected with MEC-2 cells two days prior to treatment with 370 kBq ^212^Pb-NNV003 with increasing SAs, ^212^Pb-cetuximab, NNV003 or NaCl. Mice were censored at the end of the study.

In the disseminated model of NHL, the injected Daudi cells infiltrated the bone marrow of the mice causing hind leg paralysis. The CLL model had a more aggressive profile, where the MEC-2 cells mostly infiltrated abdominal tissues, forming tumours in and around ovaries, kidneys, liver and spleen, and therefore represented a more difficult model to treat than the NHL model. Because of the aggressive profile, R2G2 mice were used for the MEC-2 model, permitting treatment with a higher dose than is possible in CB17 SCID mice. The SCID mutation impairs the DNA double strand break repair pathway, making the mouse strain inherently sensitive to radiation [35]. The mice that reached termination criteria were euthanised because of tumour infiltration or acute radiation toxicity (2 mice treated with 370 and 740 kBq ^212^Pb-NNV003). Three R2G2 mice, treated with either 185 kBq ^212^Pb-NNV003, 370 kBq ^212^Pb-cetuximab or 10 μg NNV003, and one CB17 SCID mouse treated with 185 kBq ^212^Pb-NNV003 were found dead in the cage and their cause of death was presumably related to tumour infiltration. The remaining mice were euthanised at the end of the study, 201 (Fig 5A), 197 (Fig 5B) or 150 (Fig 5C) days after cell injection.

In both models, a single injection of ^212^Pb-NNV003 significantly prolonged median survival compared to controls (Fig 5A and 5B). At study termination 28 weeks post cell injection, 67–91% of the Daudi injected CB17 SCID mice and 30–90% of the MEC-2 injected R2G2 mice treated with ^212^Pb-NNV003 were still alive. In the MEC-2 model, 555 kBq unspecific ^212^Pb-cetuximab showed an anti-tumour effect comparable to the effect of 185 kBq ^212^Pb-NNV003 (Fig 5B). There was a dose-dependent response to doses of 370 to 740 kBq ^212^Pb-NNV003, which were more effective than ^212^Pb-cetuximab, but only the 555 kBq dose of ^212^Pb-NNV003 was statistically superior.

A single i.v. injection of 370 kBq ^212^Pb-NNV003, with SA between 3.7 and 370 MBq/mg, improved survival of R2G2 mice i.v. injected with MEC-2 cells, compared to controls (Fig 5C). 60–90% of the R2G2 mice treated with ^212^Pb-NNV003 were still alive at the end of the study, 21 weeks post cell injection. No significant difference between ^212^Pb-NNV003 SAs was observed.

### Haematological toxicity of ^212^Pb-NNV003

In the R2G2 therapy study, two animals treated with 370 and 740 kBq ^212^Pb-NNV003 died 9 days post injection of suspected acute radiotoxicity. 70% of the mice in the 370 kBq group survived for more than 28 weeks with no signs of toxicity, therefore we suspect that cause of death was poisoning during grooming. Further, two mice treated with 740 kBq were euthanised due to weight loss 165 and 191 days post cell inoculation. Necropsy observations showed no macroscopic tumours; however, small spleens and pale organs might indicate radiation damage. These results indicate that 740 kBq was a too high dose and confirm the HNSTD of 555 kBq in R2G2 mice. No toxicity was observed in the CB17 SCID mice treated with 280 kBq, thus this dose was determined as HNSTD for CB17 SCID mice.

**Fig 6:**
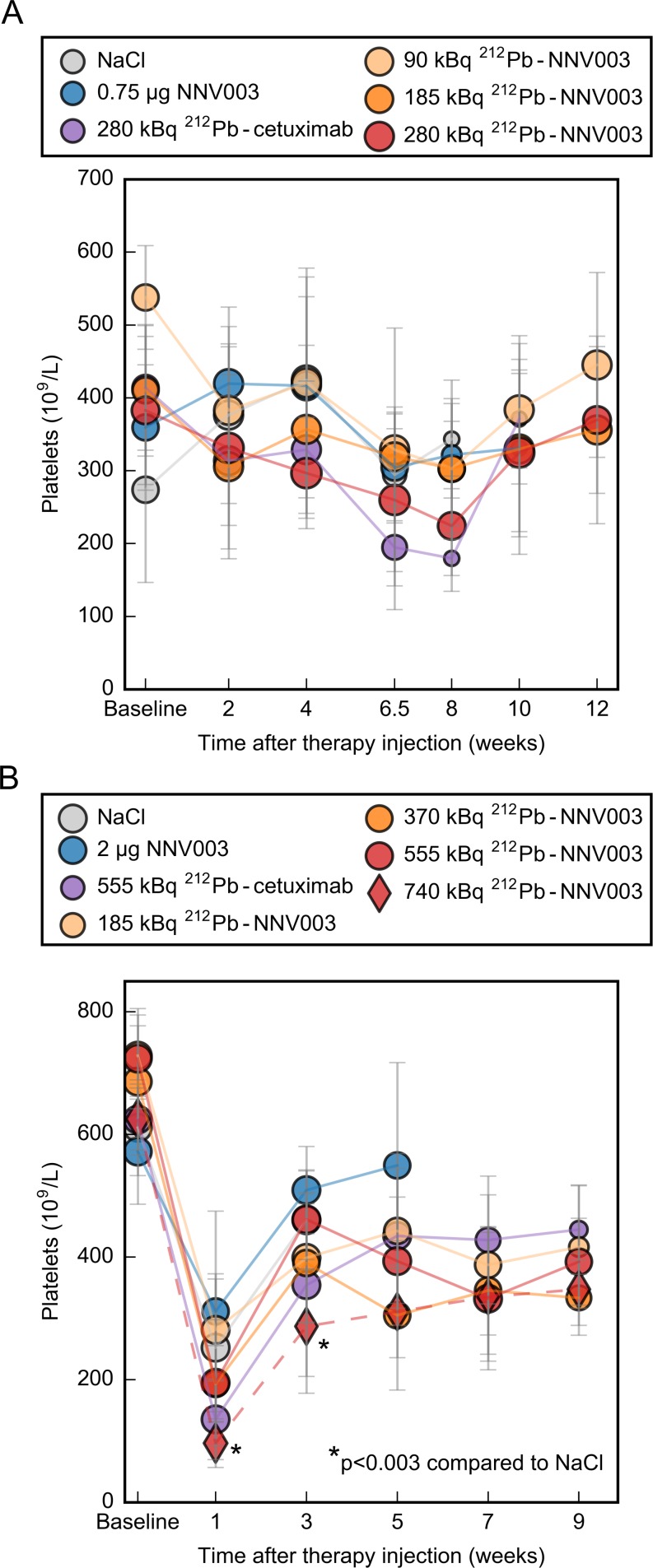
Haematological toxicity of ^212^Pb-NNV003. Platelet counts from (A) CB17 SCID mice i.v. injected with Daudi cells two days before treatment and from (B) R2G2 mice i.v. injected with MEC-2 cells two days before treatment. Average with error bars = SD. Marker size represents the number of mice at each measurement, ranging from (A) 3–11 and (B) 4–10.

At doses ranging from 185 to 555 kBq (R2G2) and 90 to 280 kBq (CB17 SCID), the haematological toxicity was modest. In R2G2 mice, but not in CB17 SCIDs, the platelet counts decreased one week after TAT injection, but only the 555 kBq treatment was significantly different from the NaCl treatment at week 1 and 3 (Fig 6A and 6B). The platelet counts also decreased in the untreated control group. We suggest this initial decrease in platelet counts to be due to the stress of being handled (three injections during three consecutive days) before blood sampling. Due to a shorter lifespan of controls, no reliable comparisons could be made after 3–4 weeks. The white blood cell- and red blood cell levels are presented in S5 Fig. Compared to controls and to baseline, no decrease in white blood cell- or red blood cell counts was observed in any of the studies.

## Discussion

For the treatment of CLL and NHL patients, new therapies with different mechanisms of actions and targets are needed to further improve outcome. In the current study, a novel anti-CD37 TAT ^212^Pb-NNV003 induced cytotoxicity in cell lines and was rapidly taken up in CD37 positive tumours. Furthermore, the TAT efficiently prolonged survival in CLL and NHL mouse models with up to 90% survival at the end of the study and low levels of haematological toxicity.

In mice injected with Daudi cells, 91% of the animals were still alive 28 weeks after receiving 90 kBq of ^212^Pb-NNV003. In the MEC-2 model, doses of 370 kBq or more were needed to achieve similar effects. This corresponds well with the *in vitro* data showing that Daudi cells were more sensitive to ^212^Pb-NNV003 compared to MEC-2 cells. Further, Daudi cells had higher and more homogeneous expression of CD37 than MEC-2 cells. MEC-2 cells present a more aggressive and invasive growth *in vivo*. Mice i.v. injected with MEC-2 cells were often euthanised due to weight loss after massive infiltration in several critical organs, while mice injected with Daudi cells were euthanised due to hind leg paralysis caused by localised infiltration of the bone marrow. Moreover, CB17 SCID mice have functional natural killer cells, whereas R2G2 do not, and the chimeric antibody NNV003 could induce some immunotherapeutic effect in this strain [8]. Accordingly, in a separate therapy study with Daudi-bearing CB17 SCID (S4 Fig) we observed that 5 μg NNV003 had a similar anti-tumour effect as 185 kBq ^212^Pb-NNV003, while 100 μg NNV003 had no effect in the MEC-2 model. Therefore, the specific activity was increased to avoid any contribution of the antibody to the therapeutic effect, and the 0.75 μg NNV003 for 280 kBq used in the present study had no effect (Fig 5A)

In theory, α-particles are more suitable than β-particles for treatment of disseminated leukemic disease due to their short range and high level of cytotoxicity. Indeed, the survival rate after 90 kBq ^212^Pb-NNV003 treatment, at less than half the HNSTD, was 91% in the Daudi model, whereas treatment with β-emitting ^177^Lu-lilotomab satetraxetan at half the HNSTD, in the same animal model, led to 10% survival 28 weeks after therapy injection [9]. It is worth noting that lilotomab is the murine version of NNV003 and although they share the same epitope, it is not a direct comparison because of the difference in immunotherapeutic capacity [8]. Human IgG1 antibodies have been shown to bind stronger to mouse Fc receptors and induce more immunotherapeutic effect than mouse IgG1 [34]. However, the large difference between the treatments cannot be explained by this as the NNV003 dose was too low to have significant anti-tumour effect. This comparison supports the theory that α-particles are more advantageous against disseminated leukemic diseases.

The effect of different SAs was investigated to select a SA for a clinical trial. A clinically relevant SA is expected to be 3.7–7.4 MBq/mg, assuming that binding of 3–4 ^212^Pb-NNV003 molecules is sufficient to eradicate a targeted cell [14]. In a phase 1 study of ^212^Pb-TCMC-trastuzumab treatment, a single i.p. infusion of up to 27.4 MBq/m^2^ was well tolerated [29], corresponding to approximately 50 MBq per patient. In our calculations we have assumed 5x10^6^ B-lymphocytes/ml in a patient [36], 5 l blood and a CD37 expression of 1x10^5^ antigens per cell [8]. An injection of 50 MBq ^212^Pb-NNV003 with the SA of 3.7 MBq/mg (corresponding to 13.5 mg ^212^Pb-NNV003), would lead to over 2x10^6^ NNV003 molecules per cell and we can thus assume 100% occupancy of the antigen. The SA of 3.7 MBq/mg means that there is 51x10^-6 212^Pb nuclei per NNV003 antibody, which leads to 5.1 ^212^Pb nuclei per cell. Consistently, we demonstrated that the anti-tumour effect of ^212^Pb-NNV003 was independent of SAs in the range of 3.7 to 370 MBq/mg. The increase in unlabelled NNV003 in the 370 kBq treatment did not have a negative impact on the therapeutic effect of ^212^Pb-NNV003.

The biodistribution of ^212^Pb-NNV003 did not reveal any unexpected accumulation in normal organs and was similar to biodistributions of other ^212^Pb labelled antibodies i.v. injected in mice. The uptake of ^212^Pb-NNV003 in liver and kidneys is consistent with results from other studies [24, 37], while the accumulation in spleen of ^212^Pb-NNV003 is lower than has been measured for another ^212^Pb labelled antibody [24]. The accumulation of ^212^Pb-NNV003 reached maximum after 24 h, resulting in an absorbed dose of 9.1 and 6.2 in the Daudi and MEC-2 tumours, respectively. A more rapid tumour targeting is expected in the i.v. models since the tumour cells are more accessible, and therefore a higher absorbed dose.

In the MEC-2 model, a modest anti-tumour effect was observed with the ^212^Pb-labeled cetuximab treatment. Cetuximab does not bind to MEC-2 cells (S1 Fig). Thus, we speculate that the observed effect may be related to the co-localisation of tumour cells in blood-rich organs. MEC-2 cells infiltrated mostly organs with a high flow of radioactive blood in the hours after injection. Especially cells localised in the kidneys would be expected to receive a significant dose due to renal excretion of the TAT.

Female mice were used in these studies for practical reasons. Although they are less prone to kidney injuries than male mice, they are more sensitive to haematological toxicity of ionising radiation, and therefore represent the worst case scenario [38, 39]. As with other TATs for treatment of diseases with bone marrow involvement, haematological toxicity was expected to be dose limiting for ^212^Pb-NNV003 [40–43]. A modest thrombocytopenia was observed in the MEC-2 model, but not in the Daudi model. No reduction in white blood cells or red blood cells was observed (S5 Fig). However, the white blood cell count in these immune deficient mice is generally lower at baseline than in other immunocompetent strains and are thus not well suited for monitoring haematological toxicity [44]. Furthermore, the total dose to femoral bone was low in both models, which might explain the modest haematological toxicity observed. However, NNV003 does not bind to murine CD37 and hence only non-specific binding of ^212^Pb-NNV003 will contribute to the absorbed radiation dose in the mice.

Around 35% of the gamma rays from ^212^Pb decay to ^212^Bi are internally converted, which can cause a ^212^Bi-chelate complex to become unstable, and when using DOTA, 30% of the ^212^Bi is released [45]. When using TCMC, 16% of ^212^Bi is released (publication in preparation). If the released ^212^Bi is circulating in the blood, it could have enough time to accumulate in the kidneys prior to decay, which could potentially be a source of non-targeted toxicity. Some indication of late radiation toxicity was observed in the 740 kBq group in the MEC-2 study, which might be due to this effect. However, the absorbed radiation doses to the kidneys at the HNSTDs (HNSTD based on haematological toxicity) were 2.5 Gy and 5.1 Gy (corresponding to 9.1 Gy/MBq, Fig 3), which is half of the dose that was found acceptable in a study with the alpha-emitter ^211^At-MX35-F(ab’)_2_ [46].

The IRF of ^212^Pb-NNV003 to CD37 was not measured before the studies were initiated and was probably around 50–60% based on post study measurements. The addition of ascorbic acid during labelling lead to an optimal IRF of around 80%, indicating that the problem was due to radiation induced oxidation and not due to the conjugation method. The IRF of ^212^Pb-NNV003 did not seem to affect the efficacy of the TAT since the therapy studies showed long term efficacy even with a suboptimal binding, and a significantly higher anti-tumour effect than the non-binding control ^212^Pb-cetuximab. We hypothesise that the increase in specific binding would lead to a better effect at lower doses of ^212^Pb-NNV003 and studies have been initiated to confirm this.

We have shown that ^212^Pb-NNV003 is effective and has a favourable safety profile in preclinical models of CD37 positive CLL and NHL. Future clinical testing is warranted.

## Supporting information

S1 File Supplementary material. Detailed description of animal models and supplementary method description.
(PDF)

S2 File ARRIVE guidelines checklist. (PDF)

S3 File Raw data. (XLSX)

S1 Table Experimental animal models. Strain, age and average weight of experimental animals at the start of the studies.
(PDF)

S1 Fig Binding of fluorescently labelled cetuximab. Flow cytometry histograms of cells (autofluorescence), cells blocked with unlabelled cetuximab and incubated with fluorescently labelled cetuximab (unspecific binding) and cells incubated with only fluorescently labelled cetuximab (total binding).
(TIF)

S2 Fig Cytotoxic effect of ^212^Pb-NNV003. Proliferation of Daudi and MEC-2 cells treated with ^212^Pb-NNV003 or ^212^Pb-cetuximab. Data represented as average of n = 8 replicates (n = 1–8 for ^212^Pb-cetuximab) and error bars = SD.
(TIF)

S3 Fig Biodistribution of ^212^Pb-NNV003 with or without IgG2a predosing. %ID/g of ^212^Pb-NNV003 in tissues of (A) CB17 SCID or (B) Balb/c mice with or without IgG2a predosing. n = 3 (no predosing Balb/c at 4 hours) or n = 5 (all other groups). Data presented as averages with error bars = SD, R = right, L = left, LPN = Lymph Node.
(TIF)

S4 Fig Anti-tumour effect of ^212^Pb-NNV003. Survival of CB17 SCID mice (n = 10 or 20) i.v. injected with Daudi cells two days prior to treatment with ^212^Pb-NNV003 (37 MBq/mg), ^212^Pb-cetuximab, NNV003 or NaCl. Mice were censored at the end of the study.
(TIF)

S5 Fig Haematological toxicity of ^212^Pb-NNV003. White blood cell counts (A and C) and red blood cell counts (B and D), measured in CB17 SCID mice i.v. injected with Daudi cells (A and B) and R2G2 mice i.v. injected with MEC-2 cells (C and D). There were 10–11 mice in each group at baseline. Marker size represents the number of mice at each measurement. Data is presented as average with error bars = SD.
(TIF)
