# Supplementary material for: Targeted alpha therapy for chronic lymphocytic leukaemia and non-Hodgkin’s lymphoma with the anti-CD37 radioimmunoconjugate 212Pb-NNV003
Source: PLoS One. 2020 Mar 18;15(3):e0230526. doi: 10.1371/journal.pone.0230526 (PMC7080250; doi:10.1371/journal.pone.0230526)
Supplement: S1 File — Detailed description of animal models and supplementary method description. (PDF) [file pone.0230526.s001.pdf]

Supplementary Material for
Targeted alpha therapy for chronic lymphocytic leukaemia and non-Hodgkin's lymphoma with the anti-CD37 radioimmunoconjugate ^{212}Pb-NNV003

Experimental animal models

S1 Table gives additional information about the mice used in the seven *in vivo* studies presented in the paper. In all studies, the mice were randomly divided into treatment (therapy/toxicity studies) or time-point (biodistribution) groups.

Supplemental binding data of unspecific control antibody

Binding of fluorescently labelled cetuximab (Alexa Fluor 647 Protein Labelling Kit, Thermo Fisher) to Daudi and MEC-2 cells was measured by flow cytometry (Guava easyCyte 12HT, Merck Millipore) to confirm unspecific binding. Briefly, three tubes were prepared with $0.25x10^6$ cells. 10 µg/ml fluorescently labelled cetuximab was added to two tubes and one of these was first blocked with an excess of unlabelled cetuximab. The total and unspecific binding of cetuximab to Daudi and MEC-2 are shown in S1 Fig. The specific binding of cetuximab was negligible in both cell lines.

Supplemental ^{212}Pb-NNV003 biodistribution data

To study the effect of murine IgG2a predosing on the ^{212}Pb-NNV003 biodistribution 10 CB17 SCID mice were i.v. injected with 370 kBq ^{212}Pb-NNV003 at day 1. The day before 5 of these mice had been i.p. injected with 200 µg murine IgG2a. The mice were euthanised 24 hours (n=5 in the IgG2a predose group, n=5 in the no-predosing group). Organs and tumours were harvested, weighted and the activity was measured by a calibrated gamma counter (Wizard2, Perkin Elmer, USA). The background was subtracted from the measurements and values were decay corrected. Percent injected dose/g (%ID/g) was calculated for each tissue. The results are presented in S3 Fig A.

To investigate the difference in ^{212}Pb-NNV003 biodistribution with or without IgG2a predosing in immunocompetent mice, the study was repeated in 18 Balb/c mice. The mice were i.v. injected with 370 kBq ^{212}Pb-NNV003 at day 1. The day before 10 of these mice had been i.p. injected with 200 µg murine IgG2a. The mice were euthanised 4 hours (n=5 in the IgG2a predose group, n=3 in the no-predosing group) and 24 hours (n=5 in both groups) after ^{212}Pb-NNV003 injection. The results are presented in S3 Fig B.

Supplemental ^{212}Pb-NNV003 therapy data

76 CB17 SCID mice were i.v. injected with $10x10^6$ Daudi cells two days before treatment with ^{212}Pb-NNV003 (37 MBq/mg), ^{212}Pb-cetuximab, NNV003-TCMC or 0.9 % NaCl (n=20 for ^{212}Pb-NNV003 groups and n=10 for the other groups). The animals received 200 µg murine IgG2a i.p. the day before treatment. The mice were checked daily for clinical symptoms and body weights were monitored. They were euthanised when termination criteria were met (see termination criteria), except one mouse treated with 185 kBq ^{212}Pb-NNV003 that was found dead in the cage. The cause of death was presumably tumour infiltration, as was the case for all the mice that reached termination criteria. Remaining mice were euthanised at the end of the study, 183 days after cell injection. Statistical analysis performed as described in Statistics section. The results are presented in S4 Fig.

Supplemental haematology data

Female R2G2 mice were i.v. injected with 2.5x10^6 MEC-2 cells two days prior to treatment with 185, 370, 555 or 740 kBq ^{212}Pb-NNV003 (370 MBq/mg), 555 kBq unspecific ^{212}Pb-cetuximab (370 MBq/mg), 2 µg NNV003-TCMC or 0.9 % NaCl (n=10 for all the groups). In a second animal model, female CB17 SCID mice were i.v. injected with 10x10^6 Burkitt's lymphoma Daudi cells two days before treatment with 90, 185 or 280 kBq ^{212}Pb-NNV003 (370 MBq/mg), 280 kBq unspecific ^{212}Pb-cetuximab, 0.75 µg NNV003 or 0.9 % NaCl (n=12 for 280 kBq ^{212}Pb-NNV003 and n=11 for the other groups). The concentration of red blood cells (RBC) and white blood cells (WBC) were monitored. 100 µL blood was collected from the retro-orbital sinus using potassium EDTA-coated capillaries and tubes, prior to treatment and every two weeks thereafter. The cell concentrations were determined using Vetscan HM5 hematology analyzer (Abaxis, USA). Animals received 300 µL 0.9 % NaCl with i.p. injection after blood collection. The results are presented in S5 Fig.
