# Supplementary material for: Targeted alpha therapy for chronic lymphocytic leukaemia and non-Hodgkin’s lymphoma with the anti-CD37 radioimmunoconjugate 212Pb-NNV003
Source: PLoS One. 2020 Mar 18;15(3):e0230526. doi: 10.1371/journal.pone.0230526 (PMC7080250; doi:10.1371/journal.pone.0230526)
Supplement: S1 Table — Strain, age and average weight of experimental animals at the start of the studies. (PDF) [file pone.0230526.s004.pdf]

S1 Table. Experimental animal models. Strain, age and average weight of experimental animals at the start of the studies.

Study	Strain	Source	Age range (weeks)	Weight at baseline (g) (range)
Daudi biodistribution	CB17/Icr-*Prkdc*^{scid}/IcrIcoCrl	Charles River Laboratories, USA	7-8	18.8 (17.4 - 19.7)
MEC-2 biodistribution	B6;129-*Rag2*^{tm1Fwa}*Il2rg*^{tm1Rsky}/DwlHsd	Envigo, USA	8-9	23.5 (20.6 - 26.9)
Acute toxicity	CB17/Icr-*Prkdc*^{scid}/IcrIcoCrl	Charles River Laboratories, USA	7-8	18.8 (15.6 - 23.4)
Acute toxicity	B6;129-*Rag2*^{tm1Fwa}*Il2rg*^{tm1Rsky}/DwlHsd	Envigo, USA	7-8	23.0 (19.8 - 26.6)
Daudi therapy study	CB17/Icr-*Prkdc*^{scid}/IcrIcoCrl	Charles River Laboratories, USA	7-8	19.4 (15.5 - 25.7)
MEC-2 therapy study (different doses)	B6;129-*Rag2*^{tm1Fwa}*Il2rg*^{tm1Rsky}/DwlHsd	Envigo, USA	13-14	26.5 (20.2 - 34.7)
MEC-2 therapy study (different SA)	B6;129-*Rag2*^{tm1Fwa}*Il2rg*^{tm1Rsky}/DwlHsd	Envigo, USA	9-10	24.2 (19.6 - 29.7)
Supplemental Daudi therapy study	CB17/Icr-*Prkdc*^{scid}/IcrIcoCrl	Charles River Laboratories, USA	8-9	19.6 (16.9 - 21.9)
Supplemental biodistribution study	BALB/cAnNHsd	Envigo, USA	7-8	18.3 (16.7-20.4)
Supplemental biodistribution study	CB17/Icr-*Prkdc*^{scid}/IcrIcoCrl	Charles River Laboratories, USA	7-8	18.1 (16.7-19.2)
