# Supplementary figures and images for: Targeted alpha therapy for chronic lymphocytic leukaemia and non-Hodgkin’s lymphoma with the anti-CD37 radioimmunoconjugate 212Pb-NNV003
Source: PLoS One. 2020 Mar 18;15(3):e0230526. doi: 10.1371/journal.pone.0230526 (PMC7080250; doi:10.1371/journal.pone.0230526)

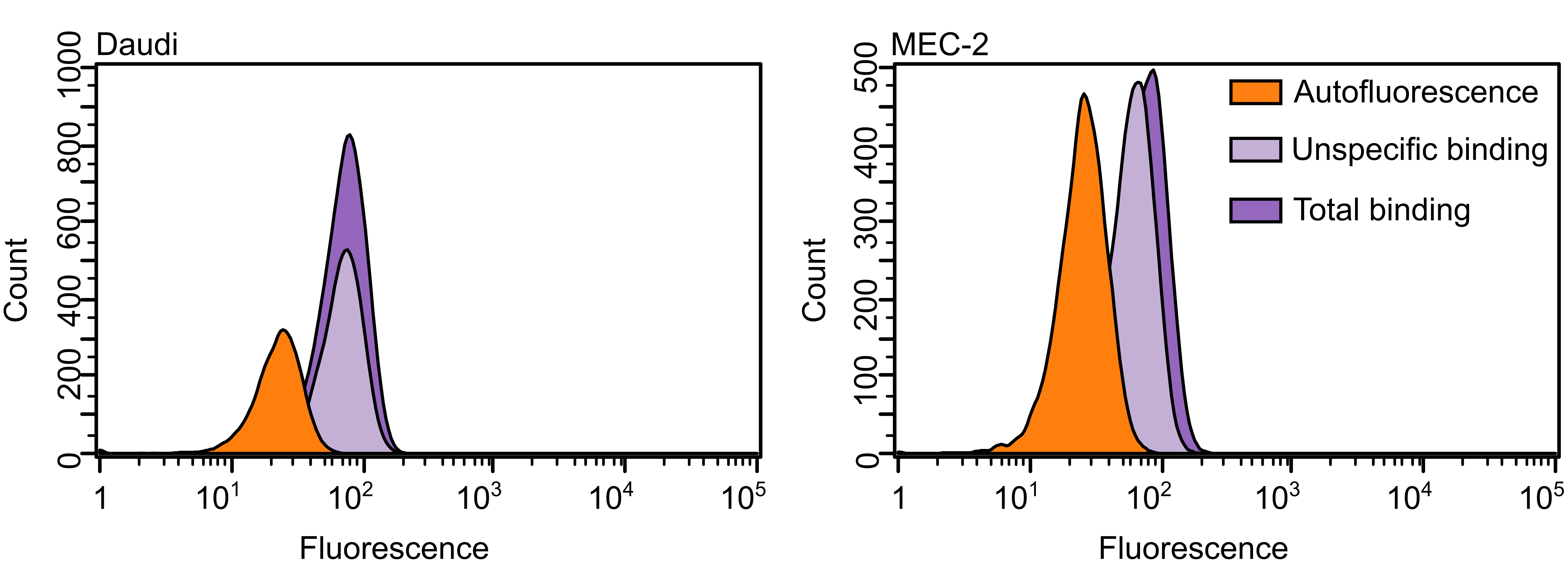

Supplement: S1 Fig — Flow cytometry histograms of cells (autofluorescence), cells blocked with unlabelled cetuximab and incubated with fluorescently labelled cetuximab (unspecific binding) and cells incubated with only fluorescently labelled cetuximab (total binding). (TIF) [file pone.0230526.s005.tif]

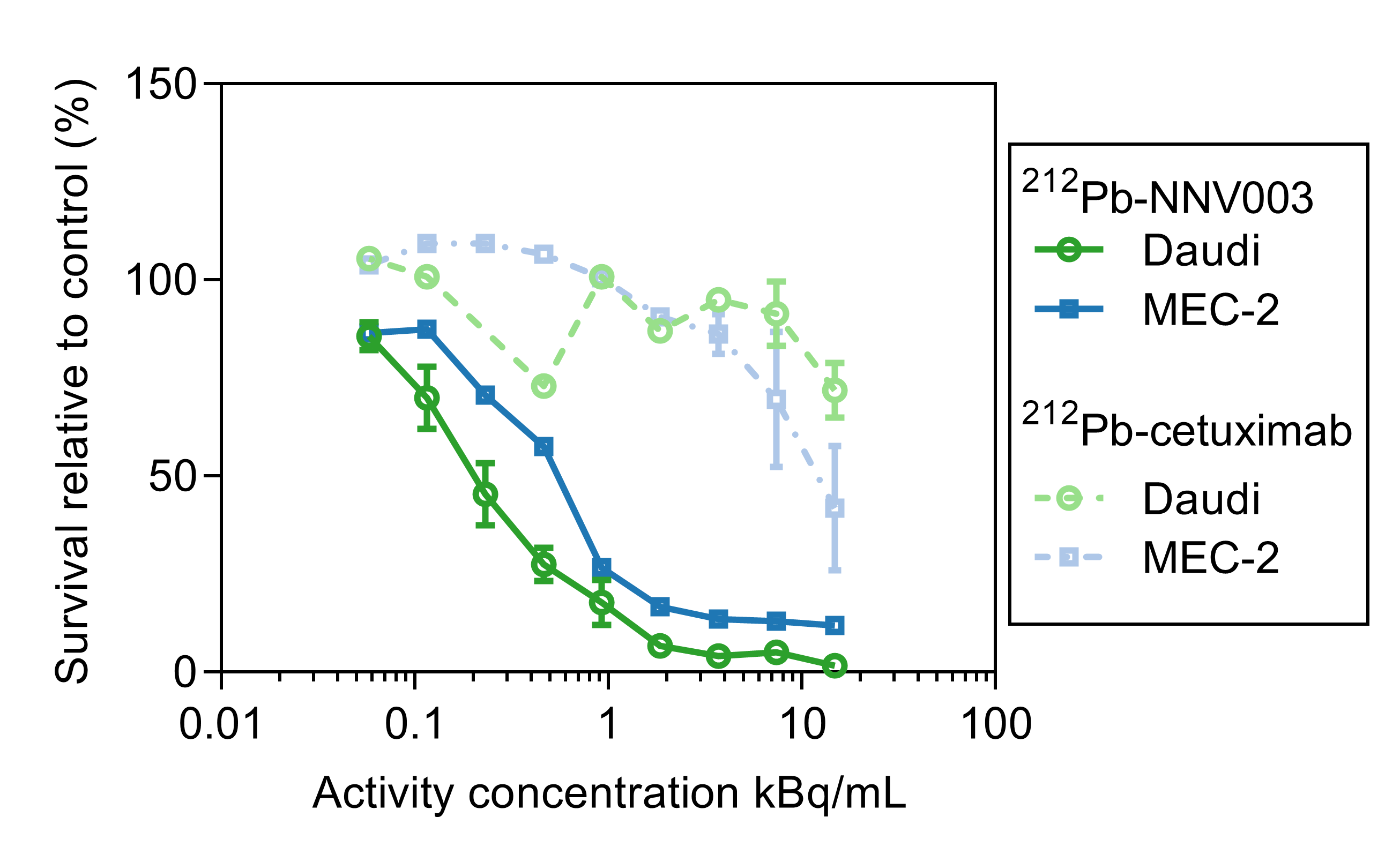

Supplement: S2 Fig — Proliferation of Daudi and MEC-2 cells treated with 212Pb-NNV003 or 212Pb-cetuximab. Data represented as average of n = 8 replicates (n = 1–8 for 212Pb-cetuximab) and error bars = SD. (TIF) [file pone.0230526.s006.tif]

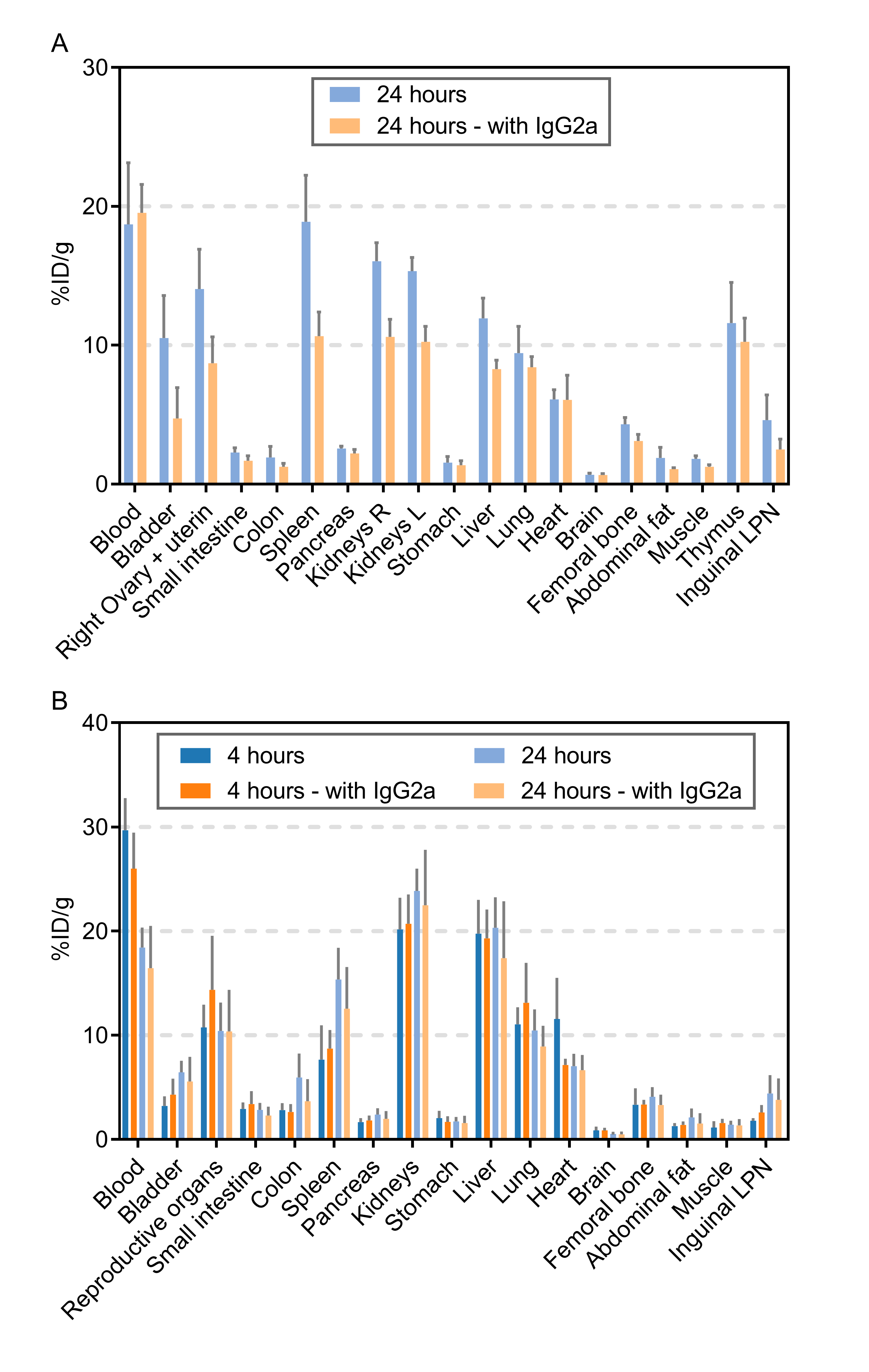

Supplement: S3 Fig — %ID/g of 212Pb-NNV003 in tissues of (A) CB17 SCID or (B) Balb/c mice with or without IgG2a predosing. n = 3 (no predosing Balb/c at 4 hours) or n = 5 (all other groups). Data presented as averages with error bars = SD, R = right, L = left, LPN = Lymph Node. (TIF) [file pone.0230526.s007.tif]

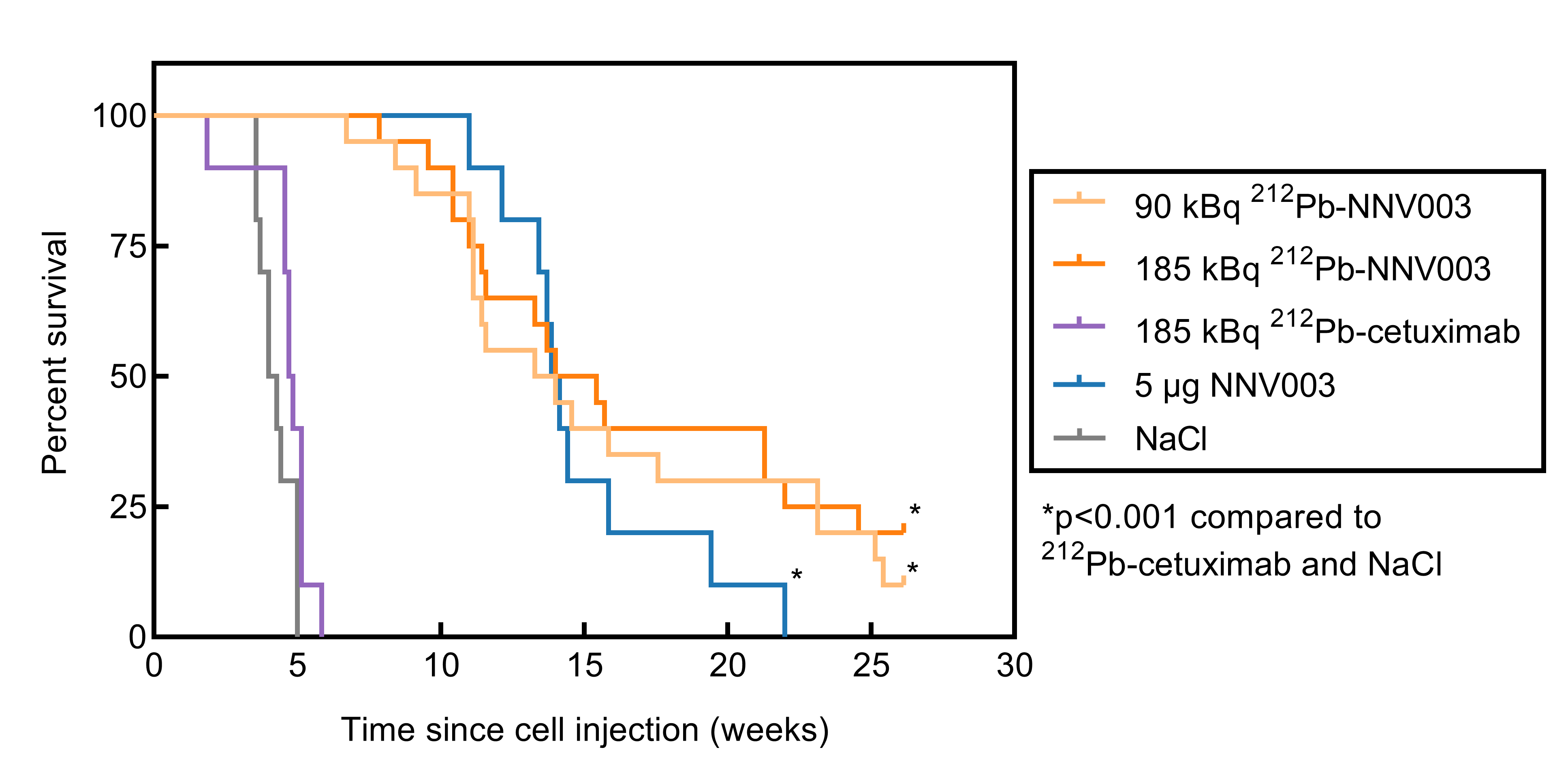

Supplement: S4 Fig — Survival of CB17 SCID mice (n = 10 or 20) i.v. injected with Daudi cells two days prior to treatment with 212Pb-NNV003 (37 MBq/mg), 212Pb-cetuximab, NNV003 or NaCl. Mice were censored at the end of the study. (TIF) [file pone.0230526.s008.tif]

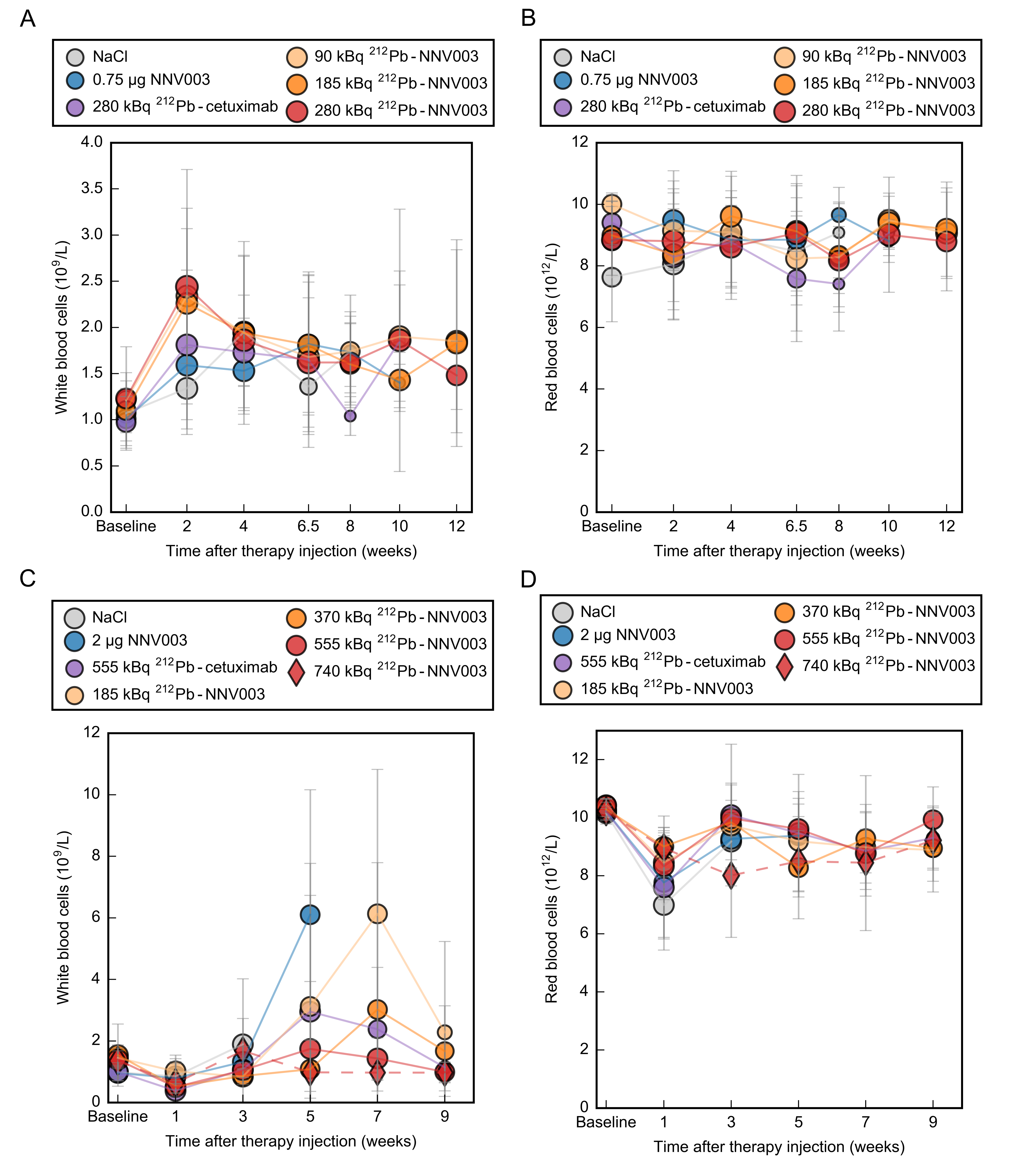

Supplement: S5 Fig — White blood cell counts (A and C) and red blood cell counts (B and D), measured in CB17 SCID mice i.v. injected with Daudi cells (A and B) and R2G2 mice i.v. injected with MEC-2 cells (C and D). There were 10–11 mice in each group at baseline. Marker size represents the number of mice at each measurement. Data is presented as average with error bars = SD. (TIF) [file pone.0230526.s009.tif]
